# Elusive Gastrointestinal Bleeding: A Rare Case of Venous Hemosuccus Pancreaticus

**DOI:** 10.7759/cureus.94118

**Published:** 2025-10-08

**Authors:** Rohan Karkra, Muhammad Hassaan A Maan, Ritik M Goyal, Sima Vossough-Teehan

**Affiliations:** 1 Department of Medicine, Rutgers University New Jersey Medical School, Newark, USA; 2 Department of Gastroenterology and Hepatology, East Orange Veterans Affairs Medical Center, East Orange, USA

**Keywords:** cancer pancreas, endoscopic ultrasound (eus), esophagogastroduodenoscopy (egd), hemosuccus pancreatic, ir guided embolization, obscure gi bleed

## Abstract

Hemosuccus pancreaticus is a rare but potentially life-threatening cause of intermittent upper gastrointestinal bleeding, characterized by hemorrhage from the pancreatic duct into the duodenum. Most cases are secondary to chronic pancreatitis and rupture of visceral artery pseudoaneurysms, particularly the splenic artery. Management typically involves CT angiography with embolization of the bleeding vessel. We describe the case of a 68-year-old male found to have spontaneous bleeding from the ampulla of Vater, later determined to be of venous origin secondary to newly diagnosed pancreatic adenocarcinoma.

## Introduction

Hemosuccus pancreaticus (HP) is a rare and potentially life-threatening cause of upper gastrointestinal hemorrhage. HP is defined as bleeding into the pancreatic duct with subsequent drainage into the duodenum via the ampulla of Vater [1]. This is typically observed in the context of chronic pancreatitis, leading to the formation of pseudoaneurysms and subsequent rupture. It can also be a sequela of pancreatic tumors, traumatic injury, or iatrogenic causes such as endoscopic retrograde cholangiopancreatography (ERCP).

HP accounts for approximately 1 in 1,500 cases of gastrointestinal hemorrhage and shows a strong male predominance, with a reported male-to-female ratio of 7:1. Classic presentations include intermittent gastrointestinal bleeding with associated epigastric pain and hyperamylasemia. Delays in diagnosis can have serious consequences, with reported mortality reaching up to 9.6% in cases that receive therapeutic interventions and 90% if left untreated [2,3].

We report the case of a male patient with chronic abdominal pain without overt signs of gastrointestinal bleeding, who was found on esophago-gastro-duodenoscopy (EGD) to have spontaneous bleeding from the ampulla of Vater. Based on diagnostic studies, bleeding was believed to be of venous origin, an exceedingly rare mechanism, which ultimately precluded the patient from undergoing any significant interventional radiology procedure.

## Case presentation

**Figure 1:**
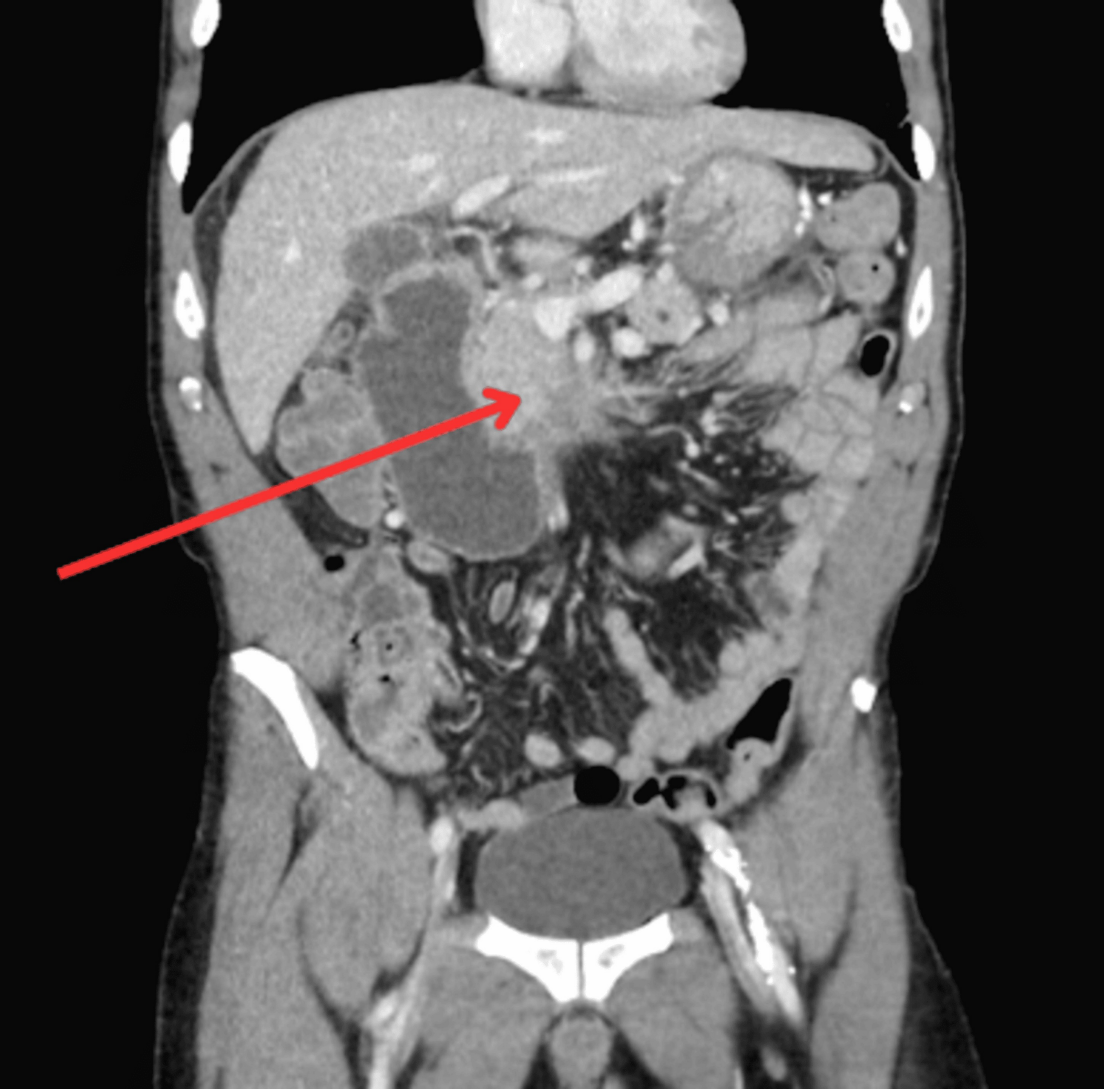
CT scan of the abdomen showing the fullness in the pancreas head with dilatation of the duodenum CT: computed tomography

A 68-year-old male with a history of tobacco use, chronic obstructive pulmonary disease, chronic pancreatitis, chronic portal vein thrombosis, and gastroesophageal reflux disease presented with two months of dull and generalized abdominal pain, early satiety, nausea, food aversion, and constipation. Laboratory investigations were notable for elevated lipase (514 U/L) and alanine aminotransferase (73 U/L). The total bilirubin, aspartate aminotransferase, alkaline phosphatase, albumin, hemoglobin (14.4 g/dl), and electrolytes were normal. A CT scan of the abdomen without contrast was obtained, which revealed fullness of the pancreatic head. Evaluation with endoscopic ultrasound (EUS) was planned, as shown in Figure 1 below.

**Figure 2:**
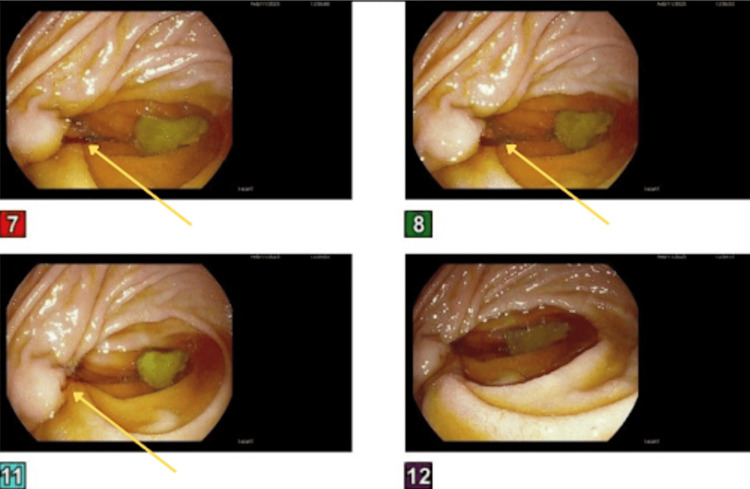
Upper endoscopy showing bleeding from the ampulla of Vater

During EGD, spontaneous bleeding was noted from the duodenal papilla/ampulla of Vater, as shown in Figure 2.

EUS showed a mass in the pancreatic head and body. Doppler did not show any obvious evidence of vascular involvement. Biopsies were obtained, but the results were inconclusive. The biopsy specimens were then sent to a reference laboratory for special staining, but the results were again inconclusive. A triple-phase CT scan obtained after the procedure again showed pancreatic and mesenteric fullness, with mild pancreatic ductal prominence (3 mm) and signs of duodenal obstruction due to extrinsic compression. Interventional radiology opined that the bleed appeared to be of venous origin and was therefore unamenable to embolization. The patient was subsequently transferred to another hospital for management of the duodenal compression. Cancer antigen (CA-19-9) was obtained, which was normal (3 U/mL). A repeat CT scan of the abdomen was obtained, which showed a 5.9 cm x 6.2 cm pancreatic head mass with 5 mm of pancreatic ductal dilatation. A repeat EGD showed gastritis, esophagitis, and partial duodenal obstruction from extrinsic compression. EUS showed a hypoechoic irregular mass in the pancreas. Biopsies were obtained, which showed moderate to poorly differentiated adenocarcinoma. A stent was deployed between the stomach and jejunum to bypass the obstruction. Oncology was consulted, and the patient was started on adjuvant chemotherapy with fluorouracil, leucovorin, irinotecan, and oxaliplatin (FOLFIRINOX).

## Discussion

The key mechanism of HP lies in the development of communication between a bleeding peripancreatic vessel or lesion and the pancreatic ductal system. In the majority of cases, this is caused by the rupture of a pseudoaneurysm of a peripancreatic artery, most commonly the splenic artery, into the pancreatic duct, often in the setting of chronic pancreatitis. Chronic inflammation and enzymatic digestion weaken the arterial wall, predisposing it to the formation of pseudoaneurysms and subsequent rupture [4]. Other etiologies include direct hemorrhage from the wall of a pseudocyst, bleeding from neoplastic lesions with ductal communication, traumatic or iatrogenic injury, and rare vascular disorders such as segmental arterial mediolysis [3,5]. It is interesting to note that in our patient, the bleed was likely venous in origin, which is exceedingly rare and not well documented in the literature. In a large case series by Yashavanth et al. [5], visceral artery aneurysms were identified in 62% of cases, with splenic artery aneurysms accounting for 37.9% of all HP presentations. Superior mesenteric artery aneurysm, pancreatic adenocarcinoma, gastrointestinal stromal tumor, post-EUS-guided fine-needle aspiration, and post-ERCP were also noted to be causes of HP [6].

The most characteristic feature of HP is intermittent upper gastrointestinal bleeding, manifesting as melena, hematemesis, or hematochezia. This intermittent nature has been attributed to cyclical clot formation and lysis within the pancreatic duct, leading to periods of clinical quiescence interspersed by episodes of overt hemorrhage. Abdominal pain, particularly in the epigastric region, is also frequently reported and may precede or accompany bleeding episodes. The classic triad of epigastric pain, gastrointestinal bleeding, and hyperamylasemia is infrequently observed in its entirety, but the presence of two of these features should at least prompt consideration of HP [1].

Yashavanth et al. [5] also noted that the median duration of bleeding prior to diagnosis was 10 days, with 40.2% of patients experiencing symptoms for more than one month, reflecting the diagnostic delay inherent to the condition. Upper gastrointestinal endoscopy is typically the initial diagnostic modality, but direct visualization of blood emanating from the ampulla of Vater is only observed in 30-65% of cases. Repeated endoscopy during active bleeding can increase the diagnostic yield. Contrast-enhanced CT, also known as multiphase CT angiography, is recommended for identifying underlying vascular lesions, pseudocysts, or neoplasms, as well as assessing for active extravasation. Selective angiography remains the gold standard for both diagnosing and localizing the bleeding source, with a diagnostic yield exceeding 85-90% when performed during active hemorrhage. It also provides a therapeutic avenue for transcatheter arterial embolization (TAE). In select cases, ERCP [7] or EUS may be helpful as adjuncts, particularly when conventional imaging is inconclusive [3,8].

The management of HP is centered on definitive control of the bleeding source and treatment of underlying pancreatic or vascular pathology. Endovascular therapy, specifically TAE, is the preferred first-line treatment for HP when a vascular etiology is identified and the patient is hemodynamically stable or can be stabilized. While TAE is minimally invasive, repeatable, and avoids the morbidity associated with pancreatic surgery, rebleeding can occur in up to 10-20% of cases, necessitating repeat embolization or surgical intervention [8,9]. Surgical management is reserved for patients in whom endovascular therapy is unsuccessful or not feasible for those with persistent or recurrent bleeding, hemodynamic instability, or associated pancreatic pathology requiring resection. Conservative management is rarely appropriate, given the risk of ongoing or recurrent hemorrhage, but may be considered in exceptional cases where the bleeding is self-limited and the underlying lesion is not amenable to intervention [10].

Due to advances in diagnostic imaging and the widespread adoption of endovascular techniques, the prognosis for HP has improved significantly. In the case series published by Lermite et al. [8], no deaths or recurrent bleeding episodes occurred during follow-up after successful intervention, whether by embolization or surgery. Long-term follow-up is, however, necessary to monitor for recurrence, particularly in patients with underlying chronic pancreatitis or persistent vascular lesions.

## Conclusions

HP is a rare but potentially life-threatening cause of upper gastrointestinal bleeding that often arises from pseudoaneurysm rupture into the pancreatic duct, most commonly in the setting of chronic pancreatitis. Early recognition is crucial, as delayed diagnosis is common due to the intermittent presentation and nonspecific symptoms. Advances in imaging and endovascular therapy, particularly TAE, have significantly improved outcomes; however, vigilant follow-up remains essential to prevent recurrence.
